# Blood Differential Gene Expression in Patients with Chronic Heart Failure and Systemic Iron Deficiency: Pathways Involved in Pathophysiology and Impact on Clinical Outcomes

**DOI:** 10.3390/jcm10214937

**Published:** 2021-10-26

**Authors:** Carles Díez-López, Marta Tajes Orduña, Cristina Enjuanes Grau, Pedro Moliner Borja, José González-Costello, Elena García-Romero, Josep Francesch Manzano, Sergi Yun Viladomat, Santiago Jiménez-Marrero, Raul Ramos-Polo, Maria del Mar Ras Jiménez, Josep Comín-Colet

**Affiliations:** 1 Bio-Heart Cardiovascular Diseases Research Group, Bellvitge Biomedical Research Institute (IDIBELL), L’Hospitalet de Llobregat, 08907 Barcelona, Spain; cdiez@bellvitgehospital.cat (C.D.-L.); mtajes@idibell.cat (M.T.O.); cenjuanes@bellvitgehospital.cat (C.E.G.); pmoliner@bellvitgehospital.cat (P.M.B.); jgonzalez@bellvitgehospital.cat (J.G.-C.); e.garcia.r@bellvitgehospital.cat (E.G.-R.); jfrancesch@idibell.cat (J.F.M.); syunvi@gmail.com (S.Y.V.); sjimenezm@bellvitgehospital.cat (S.J.-M.); raulramospolo@gmail.com (R.R.-P.); mras@bellvitgehospital.cat (M.d.M.R.J.); 2 Community Heart Failure Unit, Cardiology Department, Bellvitge University Hospital, L’Hospitalet de Llobregat, 08907 Barcelona, Spain; 3 Advanced Heart Failure and Heart Transplant Unit, Cardiology Department, Bellvitge University Hospital, L’Hospitalet de Llobregat, 08907 Barcelona, Spain; 4 Department of Clinical Sciences, School of Medicine, University of Barcelona, 08907 Barcelona, Spain; 5 Community Heart Failure Program, Cardiology Department, Bellvitge University Hospital, L’Hospitalet de Llobregat, 08907 Barcelona, Spain; 6 Department of Internal Medicine, Bellvitge University Hospital, L’Hospitalet de Llobregat, 08907 Barcelona, Spain

**Keywords:** heart failure, iron deficiency, cardiac metabolism, mitochondria

## Abstract

Background: Iron deficiency is a common disorder in patients with heart failure and is related with adverse outcomes and poor quality of life. Previous experimental studies have shown biological connections between iron homeostasis, mitochondrial metabolism, and myocardial function. However, the mechanisms involved in this crosstalk are yet to be unfolded. Methods: The present research attempts to investigate the intrinsic biological mechanisms between heart failure and iron deficiency and to identify potential prognostic biomarkers by determining the gene expression pattern in the blood of heart failure patients, using whole transcriptome and targeted TaqMan^®^ low-density array analyses. Results: We performed a stepwise cross-sectional longitudinal study in a cohort of chronic heart failure patients with and without systemic iron deficiency. First, the full transcriptome was performed in a nested case-control exploratory cohort of 7 paired patients and underscored 1128 differentially expressed transcripts according to iron status (cohort1#). Later, we analyzed the messenger RNA levels of 22 genes selected by their statistical significance and pathophysiological relevance, in a validation cohort of 71 patients (cohort 2#). Patients with systemic iron deficiency presented lower mRNA levels of mitochondrial ferritin, sirtuin-7, small integral membrane protein 20, adrenomedullin and endothelin converting enzyme-1. An intermediate mitochondrial ferritin gene expression and an intermediate or low sirtuin7 and small integral membrane protein 20 mRNA levels were associated with an increased risk of all-cause mortality and heart failure admission ((HR 2.40, 95% CI 1.04–5.50, *p*-value = 0.039), (HR 5.49, 95% CI 1.78–16.92, *p*-value = 0.003), (HR 9.51, 95% CI 2.69–33.53, *p*-value < 0.001), respectively). Conclusions: Patients with chronic heart failure present different patterns of blood gene expression depending on systemic iron status that affect pivotal genes involved in iron regulation, mitochondrial metabolism, endothelial function and cardiovascular physiology, and correlate with adverse clinical outcomes.

## 1. Introduction

Heart failure (HF) is a world-wide epidemic caused by functional and structural myocardial abnormalities that result in congestion, functional impairment and eventually, multiorgan dysfunction [1]. Contemporary medical treatment is based on the prevention of disease progression by extensive pharmacological neurohormonal blockade and device therapies [2]. Yet, despite the recent advances in the understanding of the pathophysiology of HF and the advent of new pharmacologic and device-based treatments, these patients present progressive clinical deterioration, limited life expectancy and great limitations in quality of life [3].

In the last years, iron deficiency (ID) has emerged as a key element in the current understanding of HF pathophysiology. Derangements in iron homeostasis are frequent in HF patients [4] and come along with a negative clinical impact regarding functional capacity, disease progression and mortality, independent of the hemoglobin levels [5,6,7,8]. Moreover, patients with impaired iron transport capacity (functional ID), have worse prognosis, independent of the presence of absolute ID [9,10]. Indeed, the treatment with intravenous iron has demonstrated to have a positive impact in functional capacity, quality of life and HF-related hospitalizations [11,12,13].

Nonetheless, little is known about the mechanisms by which ID occurs in HF patients, the interplay between systemic and myocardial iron status with the HF syndrome itself and the precise role of iron in myocardial performance. A combination of different clinical factors such as a reduced iron uptake, increased iron loss and impaired iron utilization explains at least part of the etiologies of ID in HF patients [14]. Recent clinical data demonstrate that patients with increased neurohormonal sympathetic activation present an impaired iron transport and increased iron demand [15], and myocardial iron content is reduced, and mitochondrial function is impaired in patients with end-stage disease [16]. Also, experimental data have disclosed part of the potential role of iron in heart function and myocardial metabolism. Iron regulation is impaired in the cardiomyocytes of patients with HF [17] and cardiomyocyte contractility is reduced in the presence of myocardial ID [18]. Moreover, recent experimental data suggest that HF neurohormonal activation induces myocardial iron depletion leading to impaired mitochondrial inducing structural and functional modifications in biological pathways in cardiomyocyte iron regulating elements that could provoke an increase in iron release and a reduction in iron uptake [19].

Despite the accumulating data, the intrinsic pathophysiological mechanisms involved in the interplay between ID and HF progression are yet to be defined. The study of ‘omics’ allows collection and characterization of diverse biological processes at different levels [20]. In this regard, the study of RNA expression (transcriptome) using high throughput technology offers a unique opportunity to massively investigate the genetic transcription and discover new mechanisms of disease [21]. Data investigating the proteomics in chronic HF patients have disclosed a few insights on the multifactorial origin of ID in patients with HF [22].

In the present study we aimed to define the biological pathways connecting ID and HF by using transcriptome analysis in a cohort of HF patients with and without systemic ID, and to explore the potential impact of genetic modifications with clinical outcomes. We hypothesized that our work might help to unveil new regulation pathways involved in intracellular and systemic iron homeostasis, to clarify its interrelation with neurohormonal activation, mitochondrial function and oxidative stress, and to identify new potential biomarkers and therapeutic targets to be used in the management of HF patients.

## 2. Materials and Methods

### 2.1. Study Design, Study Population and Ethics

The definition of the neurohormonal activation, myocardial function, genomic expression and clinical outcomes in heart failure patients (DAMOCLES) study was a single-center, observational, prospective cohort study of 1236 consecutive patients diagnosed with chronic HF recruited between January 2004 and January 2013.

The methodology of the DAMOCLES study has been published previously by our group [6,15,23,24,25,26,27,28,29]. Briefly, for inclusion, patients had to be diagnosed with chronic HF according to the European Society of Cardiology diagnostic criteria, have had at least one recent acute decompensation of HF requiring intravenous diuretic therapy (either hospitalized or in the day-care hospital), and had to be in stable condition at the time of study entry. Exclusion criteria were significant primary valvular disease, clinical signs of fluid overload, pericardial disease, restrictive cardiomyopathy, hypertrophic cardiomyopathy, hemoglobin (Hb) levels < 8.5 g/dL, active malignancy, and chronic liver disease. The patients were recruited regardless of the percentage of left ventricular ejection fraction (LVEF). In the DAMOCLES study, HF with reduced LVEF (HFrEF) was defined as LVEF ≤ 45%. The study was approved by the local committee of ethics for clinical research and was conducted in accordance with the principles of the Declaration of Helsinki. All patients gave written informed consent before study entry.

The objectives of the present study were (1) to define the pathophysiological pathways and its key biological components involved in ID in patients with chronic HFrEF and (2) explore the association of these components with clinical outcomes. First, we planned to perform a full transcriptome analysis of peripheral blood in a first cohort of patients (cohort #1) with HFrEF and ID (cases) and compare the blood RNA expression profiles of these patients with the patterns of expression in matched patients with HFrEF without ID (controls). As a second step, the differences of blood gene expression observed in the transcriptome analysis of cohort #1 were planned to be explored and replicated in a second nested case-control (ID vs. non-ID) cohort (cohort #2) of patients with HFrEF using real-time PCR. Finally, we explored the association between the gene expression of those genes significantly associated with iron status and clinical outcomes including the composite endpoint of all-cause death or HF hospitalization (primary endpoint) and all-cause death (secondary endpoint).

### 2.2. Selection Criteria and Definition of Study Cohorts

For the purpose of the present analysis, we selected 2 different nested case-control samples of patients with HFrEF. The first cohort (cohort #1) consisted in a nested-case control sample of 14 non-anemic (hemoglobin ≥ 12.5 g/dL) patients with HF (7 cases with ID and 7 matched controls without ID) from the overall DAMOCLES cohort study. The second cohort (cohort #2) was a larger nested case-control sample consisting in 71 patients with HF (35 cases with ID and 36 matched controls without ID) from the DAMOCLES study irrespective of hemoglobin levels (Hb > 9 g/dL). In both cohorts, ID and non-ID subsamples were matched according to the following variables: sex, LVEF, estimated glomerular filtration rate, etiology, hemoglobin, age and BMI.

### 2.3. Baseline Clinical Assessment

A detailed baseline evaluation was performed for all participants at study entry. This included collection of information about demographic characteristics, exhaustive medical history to gather clinical and disease related factors such as New York Heart Association (NYHA) functional class, comorbidities, laboratory information, medical treatments, and the most recent LVEF. Sources of information were the medical history and standardized questionnaires.

### 2.4. Blood Sample Management and Laboratory Assessments

Laboratory data and blood sample management methods have been previously re-ported by our group [6,10,15,24,25]. Briefly, patients were resting in a supine position in a quiet room for 30–60 min after venous cannulation. Blood samples were collected using Vacutainer^®^ (BD, Franklin Lakes, NJ, USA) and 10 mL EDTA tubes and immediately processed as follows: 4 plasma aliquots 250–500 µL each from one 10 mL EDTA tube, 4 serum aliquots 250–500 µL each from 5 mL serum Vacutainer^®^ tubes. Resulting plasma and serum aliquots were frozen and stored at −80 °C using the Micronics^®^ (Micronics Ltd., High Wycombe, Bucks, UK) system. Blood for DNA and/or RNA studies was stored in 10 mL EDTA tubes at −80 °C until being processed for extraction. Additional blood samples collected on EDTA and Vacutainer^®^ tubes were immersed in melting ice and frozen until they were processed for routine local laboratory analyses.

Serum N-terminal pro b-type natriuretic peptide (NT-proBNP) levels were measured in pg/mL using an immunoassay based on chemiluminescence using Elecsys System (Roche^®^, Basel, Switzerland). Serum iron (mg/dL) was measured using spectrophotometry; serum ferritin (ng/mL) and transferrin (mg/dL) were measured using immunoturbidimetry. Transferrin saturation (TSAT) was estimated using the formula: TSAT = serum iron (mg/dL)/(serum transferrin (mg/dL) × 1.25). Iron status was also assessed by measuring serum soluble transferrin receptor (sTfR in mg/L) levels using an enzyme immunoassay. Hb (g/dL) was measured with impedance laser colorimetry. The glomerular filtration rate (GFR) was estimated from serum creatinine using the formula of Modification of Diet in Renal Disease Study Group (MDRD equation). Iron deficiency was defined using the Kidney Disease Outcomes Quality Initiative (KDOQI) criteria which categorizes ID as ferritin < 100 ng/mL and/or TSAT < 20% [10,15].

### 2.5. RNA Extraction and Blood Gene Expression Analyses

First, total RNA was isolated from whole blood samples using Nucleospin RNA II kit (Macherey-Nagel^©^, Düren, North Rhine-Westphalia, Germany) according to the manufacturer’s recommendations. RNA was quantified by a NanoDrop 1000 Spectrophotometer (Thermo Fisher Scientific^©^, Waltham, MA, USA).

The transcriptome analyses were undertaken in the blood samples of patients from cohort#1. Transcriptome processing protocol was performed as previously described by García-Díez et al. [30] Briefly, sample processing, amplification, labeling, and hybridizations were performed according to the protocol for the GeneChip WT PLUS Reagent kit and then hybridized to GeneChip Human Gene 2.0 ST Array (Affymetrix) in a GeneChip Hybridization Oven 640 (Thermo Fisher Scientific^©^, Waltham, MA, USA). Washing and scanning were performed using the Expression Wash, Stain and Scan Kit and the GeneChip System of Affymetrix (GeneChip Fluidics Station 450 and GeneChip Scanner 3000 7G). After quality control of raw data, they were background corrected, quantile normalized and summarized to a gene level using the robust multichip average (RMA) [31], resulting in a total of 48,144 transcript clusters, which roughly corresponded to genes or other mRNAs as miRNAs or lncRNAs. Linear Models for Microarray (LIMMA) was used to detect paired differentially expressed genes between the two different samples, with a *p*-value less than 0.05 and an absolute fold change above 1.2 considered as significant. The omics data generated in this study have been deposited in NCBI’s Gene Expression Omnibus [20] and are available through GEO Series accession number GSE175739.

Based on transcriptome analyses, the 1128 transcripts that were differentially expressed in ID samples compared to controls were classified in 20 main ontology clusters using Gene Ontology (GO) and Kyoto encyclopedia of Genes and Genomes (KEGG) pathway enrichment analysis performed by Metascape [32]. Based on that classification, statistical significance and pathophysiology, 22 genes were selected to be explored in cohort #2. The 22 candidate genes were also classified in 8 main ontology clusters using GO and KEGG pathway enrichment analysis performed by Metascape.

As a second step, the RNA expression levels of the 22 candidate genes selected from transcriptome analysis were further explored in the blood samples of cohort #2 using real time (rt) quantitative Polymerase Chain Reaction (PCR) analysis by TaqMan^®^ Low Density Array (TLDA) technology (Thermo Fisher Scientific^©^, Waltham, MA, USA). Briefly, gene expression was evaluated by generating a custom-made Applied Biosystems TaqMan^®^ Low Density Array (TLDA). Accordingly, the gene expression of selected genes was assessed by TLDA in a nested case-control sample of 71 patients with HFrEF (cohort #2) consisting of 35 patients with ID and 36 patients with normal iron status. Data were normalized to 18s, and relative quantification was performed using the comparative Ct (cycle threshold) method (2-DDCt). According to this, mRNA levels of ID patients (cases) were expressed as fold change vs. control (samples without ID). In addition, we explored the relationship between individual gene expression, expressed as delta Ct values of individual genes, and clinical outcomes. Delta Ct values corresponded to the difference between CtSOI (sequence of interest) and Ct of gene 18s, our reference housekeeping gene sequence (CtRS). Delta Ct was calculated subtracting CtRS from CtSOI. This approach has been used in previous research [33,34,35].

### 2.6. Follow Up and Major Heart Failure Events Ascertainment

Follow-up in DAMOCLES lasted until November 2015. Study participants were followed for a median of 2.93 years (mean 3.3 years). Follow-up was conducted by trained study personnel. Specifically, data on mortality and on cause of death were obtained from hospital and primary care electronic medical records, and/or by direct interview with the patients’ relatives.

### 2.7. Statistical Methods

Using the baseline and follow-up data from the DAMOCLES cohort, cross-sectional and longitudinal descriptive analyses were performed. Demographic and clinical characteristics, as well as laboratory tests results were summarized using basic descriptive statistics according to iron status strata in both cohorts.

For categorical variables, number and percentage were reported, and for continuous variables, mean (standard deviation) or median (interquartile range) were used, depending on the distribution of the variables. χ^2^, Student’s T, and non-parametric tests were used to compare characteristics across strata.

In the PCR-Real time analysis, relative gene expression data from blood samples were expressed as the mean ± standard error of mean (SEM) and significant differences between strata were established by Student’s t-test.

To explore the association between RNA expression of these genes and the primary composite (all-cause death or HF hospitalization) and secondary (all-cause death) endpoints, we developed several generalized additive models (GAM). These models were designed to explore parametric and non-parametric relationships between blood gene expression, expressed as delta Ct values of individual genes, and outcomes, expressed as the beta estimates of primary and secondary endpoints. Ct levels are inversely proportional to the amount of target nucleic acid in the sample (i.e., the lower the Ct level the greater the amount of target nucleic acid in the sample). All models were adjusted for important prognostic variables.

Furthermore, these adjusted models were replicated using adjusted Cox proportional hazards analyses exploring associations of gene expression with outcomes. In these models, gene expression expressed as delta Ct values of individual genes was divided in tertiles and tertiles were grouped considering the predominance of linear or non-linear patterns of association for each gene.

All statistical tests and confidence intervals (CI) were constructed with a type I error alpha level of 5%. For transcriptome analyses, adjusted *p*-value for multiple hypothesis testing was evaluated using the Benjamini-Hochberg method. *p*-Values below 0.05 were considered statistically significant. All analyses were performed using SPSS software (version 25.0; IBM, Armonk, NY, U.S.), InStat GraphPad, and R software (versions 4.0.2 and 3.1.1; R Foundation for Statistical Computing, Vienna, Austria). Bioinformatic analyses of the transcriptome were conducted using the R packages aroma Affymetrix, Biobase and Limma [30].

## 3. Results

In total, a nested case-control sample of 14 patients with HF (7 cases with ID and 7 matched controls without ID) from the DAMOCLES study defined cohort #1 (transcriptome cohort). Equally, a larger nested case-control sample of 71 patients with HF (35 cases with ID and 36 matched controls without ID) from the DAMOCLES study defined cohort #2 (rt-PCR evaluation cohort).

As shown in Table 1, study groups in both cohorts were well balanced according to baseline characteristics other than iron status. One third of patients were female, most of patients where in NYHA functional class I or II, mean LVEF was below 40%, and the most common HF etiology was ischemic cardiomyopathy. No between-groups differences were found regarding the use of beta-blockers, angiotensin receptor antagonists or diuretics in any of the cohorts. Finally, the prevalence of common HF comorbidities was similar between ID and non-ID patients, although in cohort 2# patients with ID where more frequently diabetic when compared to patients without ID (28 (77.8%) vs. 18 (51.5%); *p*: 0.002). A similar trend was found in Cohort 1# (5 (71.4% vs. 2 (28.6%) *p*: 0.286)).

The peripheral blood samples from cohort #1 underwent a full transcriptome analysis to study the differential transcripts levels between both populations. The first analysis of the whole transcriptome highlighted 1128 differentially expressed transcripts. From all these transcripts, we performed an enriched gene analysis of the differentially expressed genes from the transcriptome study using Metascape (Supplementary Figure S1). Statistical tests for multiple hypothesis testing were not significant. Among the unadjusted analyses of the transcripts, 22 genes involved in deranged metabolic pathways in HF, cellular iron regulation and metabolism were selected for further evaluation according to statistical significance and/or pathophysiological relevance.

**Figure 1:**
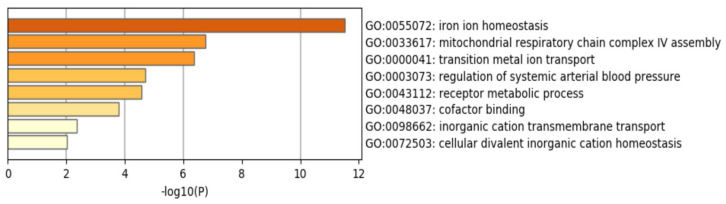
Enriched gene ontology (GO) terms of the 22 candidate genes evaluated in cohort #2. Analysis was carried out by Metascape. The x-axis denotes −log10 (*p*) values based on the cumulative hypergeometric distributions. The colors denote de relative value of −log10 (*p*): darker colors indicate a greater value of −log10 (*p*).

Table 2 presents the description of the 22 candidates genes selected to from transcriptome analysis of cohort #1. Enriched gene ontology of the 22 selected candidate genes is presented in Figure 1. Briefly, the selected genes were important components of HF neurohormonal axis, iron homeostasis and mitochondrial metabolism.

Finally, to deepen our understanding of these metabolic pathways and corroborate our previous results, we used TaqMan low-density array (TLDA) cards for real-time PCR detection (TLDA PCR) tests, to measure the RNA expression of these 22 genes and evaluated their association with ID status in a larger cohort of patients from the DAMOCLES study (cohort 2#).

**Figure 2:**
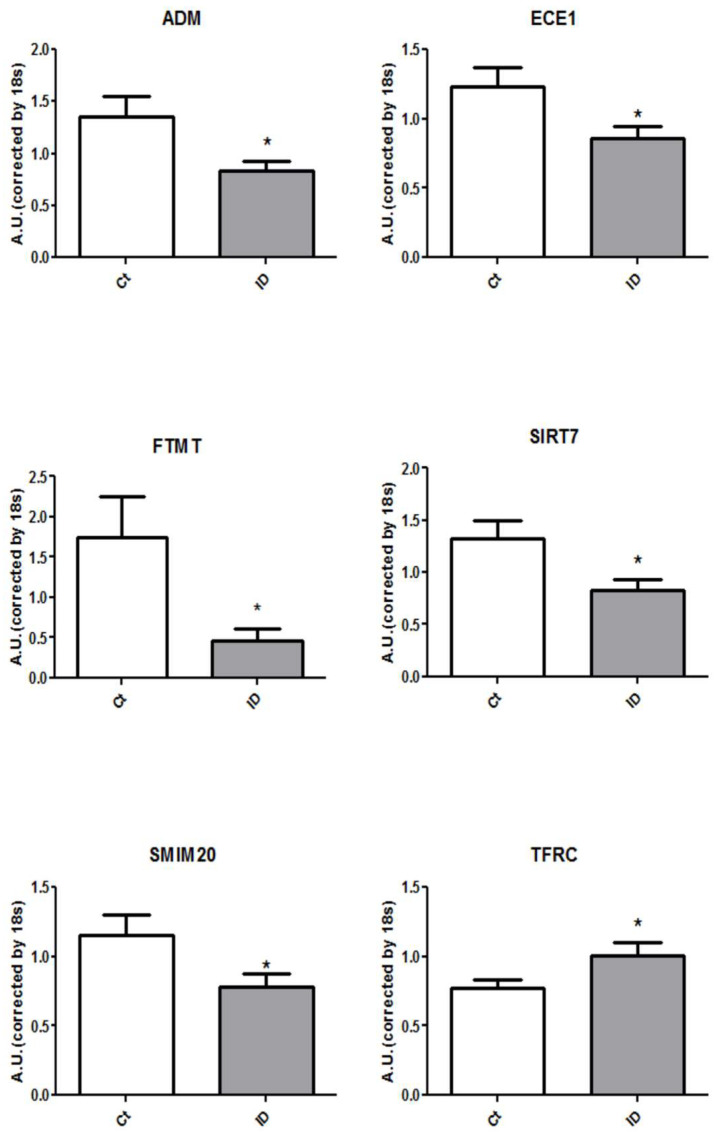
*ADM*, *ECE1*, *FTMT*, *SIRT7*, *SMIM20* and *TFRC* genes are differentially expressed in the whole blood of ID (iron deficiency) patients versus the control ones. Graph representation of the 6 mRNA levels from blood in both studied populations. Data were normalized by the 18 s and expressed as mean ± SEM (* *p* < 0.05 vs. Control).

As shown in Table 3 and Figure 2, only 6 genes showed significant differences in gene expression depending on iron status: transferrin receptor (TFRC), mitochondrial ferritin (FTMT), sirtuin 7 (SIRT7), small integral membrane protein 20 (SMIM20), adrenomedullin (ADM) and endothelin converting enzyme 1 (ECE1). The RNA expressions of FTMF, SIRT7, SMIM20, ADM and ECE1 were significantly reduced in blood samples of patients with systemic ID compared to control patients with normal iron status. Fold changes in mRNA levels comparing ID vs. non-ID patients ranged from 0.62 to 0.70 (all *p*-values < 0.05). On the other hand, patients with systemic ID showed an increased ex-pression of TFRC with a statistically significant (*p*-value 0.03) 1.31 increased fold change in RNA expression compared to control patients without ID.

**Figure 3:**
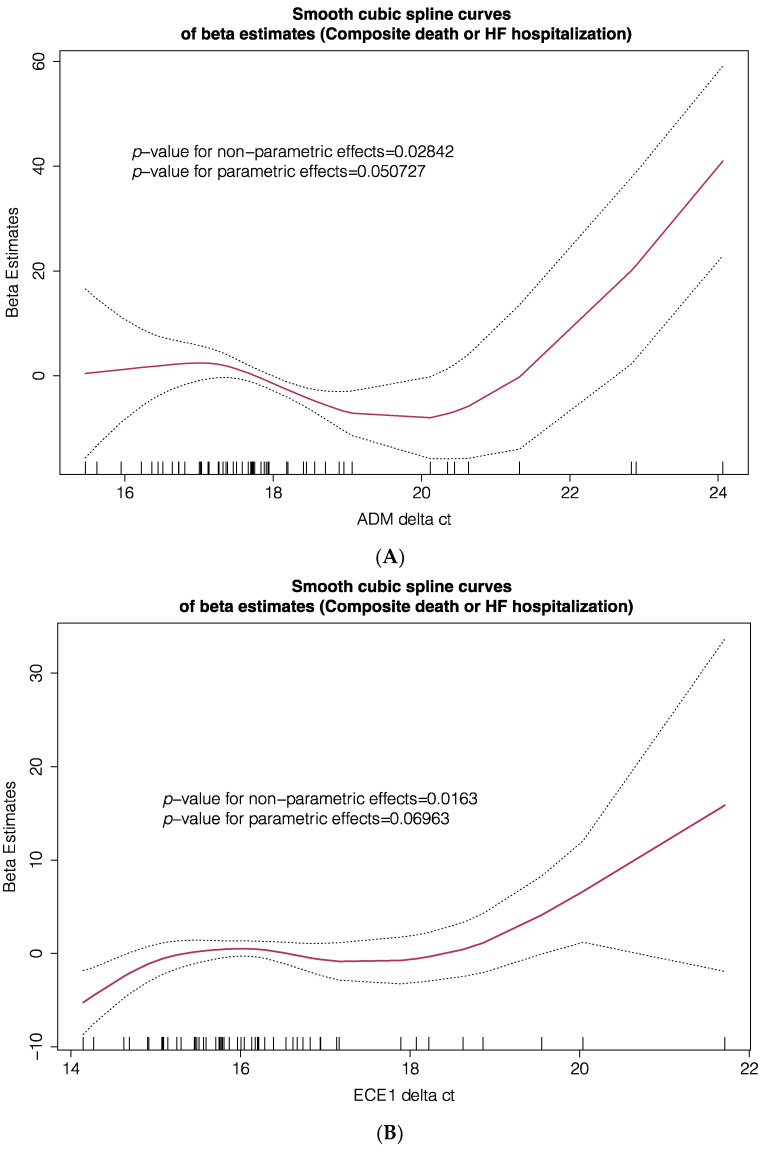

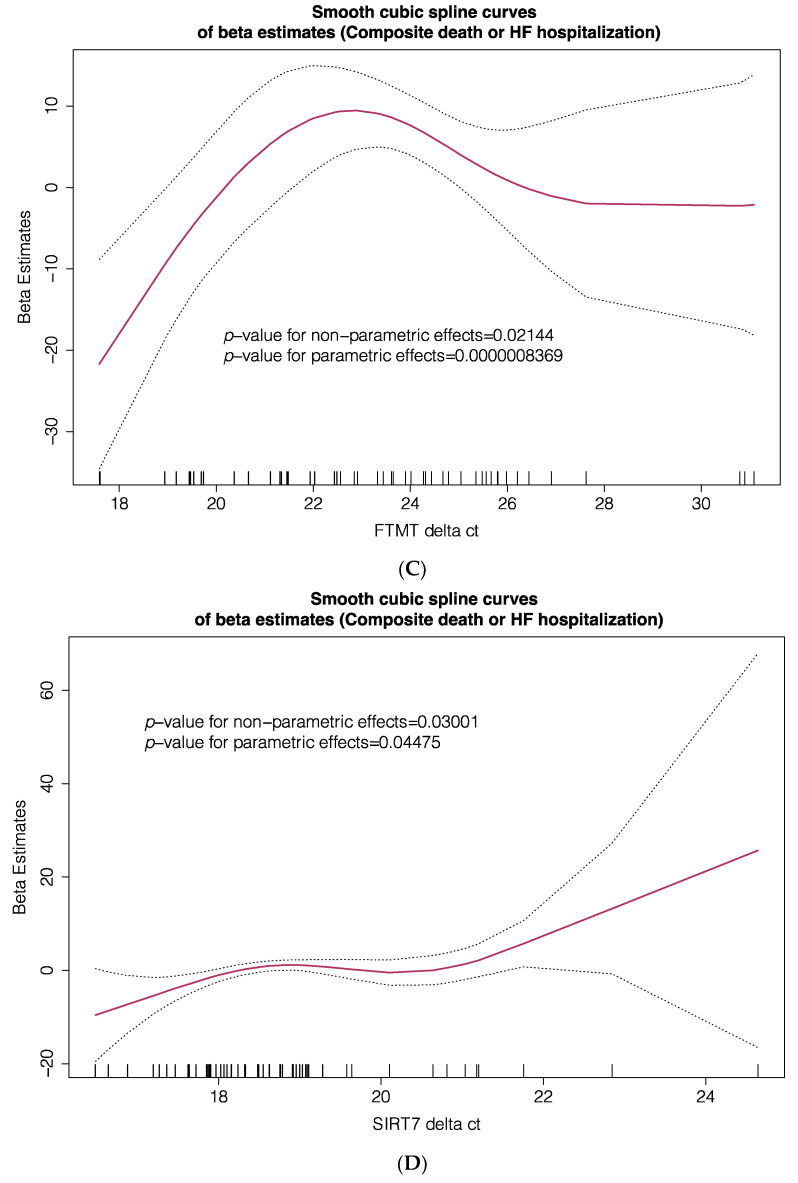

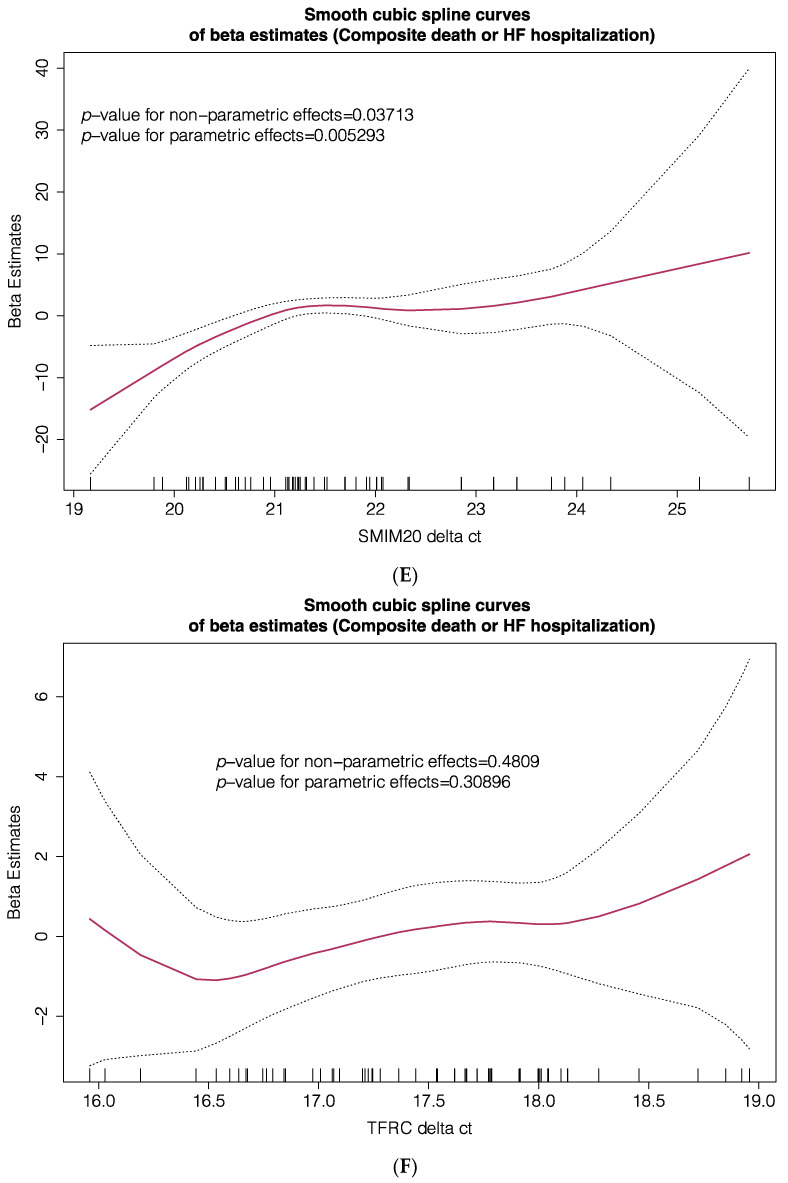
Smooth spline estimates of the composite endpoint (all-cause death or HF hospitalization) according to gene expression of *ADM* (**A**), *ECE1* (**B**), *FTMT* (**C**), *SIRT7* (**D**), *SMIM20* (**E**) and *TFRC* (**F**). Δct: change in gene expression cycle threshold). Ct levels are inversely proportional to the amount of target nucleic acid in the sample (i.e., the lower the Ct level the greater the amount of target nucleic acid in the sample). Plots show the beta estimates of the outcome.

To further explore the prognostic clinical implications of these 6 differentially expressed genes, we evaluated the associations between their mRNA expression and the primary composite (all-cause death or HF hospitalization) and secondary (all-cause death) endpoints, by developing several generalized additive models (GAM). These models were designed to explore parametric and non-parametric relationships between blood gene expression, expressed as delta Ct values of individual genes, and outcomes, expressed as the beta estimates of primary and secondary endpoints. Models were adjusted for important prognostic variables including age, gender, left ventricular ejection fraction, use of disease-modifying drugs, comorbidities, functional capacity, natriuretic peptide levels and renal function. As a result of these multivariable GAM, smooth spline estimates of the primary composite endpoint (all-cause death or HF hospitalization, Figure 3) and the secondary endpoint (all-cause death, Supplementary Figure S2) according to blood gene expression of ADM, ECE1, FTMT, SIRT7, SMIM20 and TFRC were plotted. Significant linear and non-linear relationships between gene expression and the primary endpoint were found for ADM, ECE1, FTMT, SIRT7, SMIM20 but not for TFRC. These associations were also confirmed for the secondary endpoint only for FTMT and SMIM20.

Furthermore, these models were replicated using adjusted Cox proportional hazards analyses exploring associations of gene expression with outcomes. Gene expression was divided in tertiles and tertiles were grouped in categories. To improve clinical interpretation of the results, grouping was conducted considering the predominance of linear or non-linear patterns of association for each gene. As shown in Table 4 and Supplementary Figure S3, intermediate FTMT gene expression compared to high or low FTMT gene expression was associated with and increased risk of the primary endpoint (HR 2.40, 95% CI 1.04–5.50, *p*-value = 0.039). Similar results were observed for intermediate or low SIRT7 gene expression compared to high SIRT7 gene expression (HR 5.49, 95% CI 1.78–16.92, *p*-value = 0.003) and for intermediate or low SMIM20 gene expression compared to high SMIM20 gene expression (HR 9.51, 95% CI 2.69–33.53, *p*-value < 0.001).

Moreover, as presented in Table 4 and Supplementary Figure S4, the association of intermediate FTMT gene expression and intermediate or low SIRT7 or SMIM20 gene expressions with all-cause death was also confirmed in multivariable analyses. Cox proportional hazards model analyses did not confirm associations with outcomes for the remaining genes studied for any of the outcomes.

## 4. Discussion

This is the first study to find consistent gene transcription differences in key functional pathways that connect iron homeostasis with mitochondrial function, oxidative stress and cardiovascular physiology, in patients with chronic HF. Moreover, we found an association between these transcriptomic signals and prognosis.

HF is a complex multisystemic disease involving numerous biological processes that influence the myocardial structure and function [36,37]. Despite optimal gold-standard treatment, a significant number of patients present higher clinical deterioration [3], suggesting that there are unknown biologic components playing a role in disease progression beyond the typical neurohormonal activation. In this scenario, systemic and myocardial iron homeostasis have emerged as relevant factors that participate in the HF syndrome given that: (1) a significant number of HF patients have systemic ID [4], (2) patients with ID have a worse clinical course when compared with non-ID patients [5,6,8,25], and (3) treatment of ID patients with intravenous iron results in significant clinical improvements [12,13,38,39].

Limited investigational data have disclosed a few of the mechanisms that connect HF and ID. From a pathophysiological point of view, clinical data indicate that ID in HF may be related to low intake, gut losses, persistent congestion, chronic kidney disease, malnourishment and inflammation [22]. In this regard, functional ID has been shown to be one of the main mechanisms that explain ID at a tissue level and to correlate with prognosis [9,10]. Our group has been long investigating the relationship between HF neurohormonal activation and systemic and myocardial iron homeostasis. We have previously demonstrated that patients with ID presented higher noradrenaline levels and that this increased neurohormonal activation was related with impaired iron homeostasis [15]. Moreover, we have recently demonstrated that key iron regulating elements were altered in mice with HF, leading to myocardial ID, and we have shown that the stimulation of cardiac cells with norepinephrine and angiotensin, altered critical iron regulation components, generating intracellular ID and mitochondrial dysfunction [19].

In the present study, we exposed some of these biological crosstalks by analyzing the transcriptomic footprint in a two-phase stepwise cross-sectional study using two nested case control matched cohorts with and without ID: the exploratory cohort (cohort1 #) and the validation cohort (cohort 2#). Full transcriptome results underscored clusters of genes related to myocardial metabolism, iron homeostasis and cardiovascular physiology that were subject to the iron status. Among these, we selected 22 candidate genes according to the highlighted biological pathways by the enriched gene ontology analyses, statistical significance and biological interest, whose expression levels were subsequently analyzed by rt-PCR analyses in the validation cohort 2#. In this cohort, we found that patients with ID presented a lower expression of FTMT, SIRT7, SMIM20, ADM and ECE1 genes compared to non-ID patients, confirming the initial insights outlined in the exploratory transcriptome. Furthermore, in multivariable regression analyses, we found a statistically significant correlation between the levels of FTMT, SIRT7 and SMIM20 gene expression, with the risk of all-cause death and HF hospitalizations.

Transcriptome analyses constitute both a challenge and an opportunity to better understand the mechanistic insights of diseases and a gateway to discover new biomarkers and treatments to improve the management of patients. Indeed, the dysregulation of both coding and non-coding RNA are important elements in cardiac physiology [40,41,42]. In HF, RNA high throughput analyses have underscored the importance of mRNA in cardiac homeostasis tackling the control of the vascular system, inflammation and cardiac regeneration [21], and previous works have shown that the analysis of specific mRNA might have prognostic implications in HF patients [43]. Similarly, the analysis of proteomic profiles in HF patients have emphasized the importance of these metabolic pathways [44,45].

In our cohort of chronic HF patients, we found that HF and ID are indeed connected in a way that patients with systemic ID have a significant dysregulation in the ex-pression of pivotal genes related with iron regulation, mitochondrial metabolism, endothelial function and neurohormonal activation. Moreover, we found that an intermediate FTMT gene expression and intermediate or low SIRT7 or SMIM20 gene expressions were related with worse outcomes. Cardiac metabolism and mitochondrial function are critical elements in HF progression and a matter of study for future treatments [46,47]. In this regard, SIRT7 gene is a member of the mammalian sirtuins, a group of NAD + -dependent deacylases with roles in genes transcription and DNA repair, which are responsible for the control of oxidative stress, energy metabolism and cellular senescence [48]. From a cardiovascular point of view, sirtuins are known to participate in lipid metabolism and endothelial function, and to protect from inflammation, the development of atherosclerosis, left ventricular hypertrophy and fibrosis [49,50,51]. SMIM20 is situated in the inner mitochondrial membrane and takes part of the regulation of the cytochrome c oxidase complex [52] and in the heart, it is thought to play a cardioprotective role [53]. Finally, FTMT acts as an antioxidant in the mitochondrial matrix and has a critical role in the management of iron and DNA repair [54].

Together, the results of our study point to genetic transcription differences in ID patients that affect critical elements of pathophysiological pathways that involve iron regulation, mitochondrial function, oxidative stress and cardiovascular physiology. These findings demonstrate the biological interplay between ID, HF typical neurohormonal activation and mitochondrial metabolism as key elements that might help in the understanding of the underlaying mechanisms that participate in HF progression. Moreover, the prognostic relevance of our findings regarding these gene transcription modifications paves the way to the potential use of these critical metabolic pathways as biomarkers of mitochondrial dysfunction and oxidative stress, and its use as a target for future treatments.

Our study has several limitations to be acknowledged. First, this study was a single-center study and hence, our findings may not be representative of all HF patients. Second, we performed a cross-sectional study and therefore, the statistical correlations that we found merely point to an association between ID and the differences in transcriptomics. Indeed, although our statistical findings suggest that patients with ID presented derangements in the metabolic pathways that implicate mitochondrial function, oxidative stress and iron regulation, it could also be possible that such gene transcription differences caused systemic ID and that the prognostic implications found indicated that patients were in a sicker condition. Third, as we have demonstrated previously, the pathophysiological pathways involving ID, mitochondrial function and HF are multiple and therefore, the genes underscored by our analyses are only a part of a wider complex network. Finally, by using the standard definition of ID we indirectly estimated the true iron status of patients, yet this is the most common method used daily practice.

## 5. Conclusions

In patients with chronic HF, we determined differences in the transcription of genes according to the presence of ID that affect the pathophysiological pathways of mitochondrial metabolism, iron regulation and cardiovascular physiology. Future research may confirm the prognostic implications found in the transcription levels of FTMT, SIRT7 and SMIM20 genes. These new insights underscore the importance of iron homoeostasis in the interplay between heart metabolism and myocardial dysfunction.

## Figures and Tables

**Table 1:** Characteristics of cohorts included into the study. Cohort #1 (*n* = 14, transcriptome analysis), Cohort #2 (*n* = 71, TLDA-PCR).

	Cohort #1 (Transcriptome) *n* = 14			Cohort #2 (TLDA PCR) *n* = 71		
	ID	Non-ID		ID	Non-ID	
	*N* = 7	*N* = 7	*P*-Value	*N* = 35	*N* = 36	*p*-Value
**Demographic and Clinical Factors**						
Age, years	70 (9)	71 (10)	0.762	70 (12)	72 (11)	0.472
Sex (male), *n* (%)	6 (86)	6 (86)	1.0	23 (64)	22 (63)	1.0
Systolic blood pressure, mmHg	135 (19)	124 (22)	0.309	123 (20)	120 (17)	0.495
**NYHA functional class**						
I-II	5 (71)	5 (71)	1.0			
III-IV	2 (29)	2 (29)				
LVEF, %	36 (5)	35 (6)	0.772	33 (7)	34 (6)	0.908
Ischemic etiology of HF, *n* (%)	5 (71)	5 (71)	1.0	22 (61)	18 (51)	0.477
6 mwt distance	248 (138)	287 (155)	0.618	230 (141)	257 (172)	0.488
**Comorbidities**						
Hypertension, *n* (%)	6 (86)	6 (86)	1.0	29 (81)	27 (77)	0.778
Diabetes Mellitus, *n* (%)	5 (71)	2 (29)	0.286	28 (78)	18 (51)	0.026
Previous MI, *n* (%)	2 (29)	2 (29)	1.0	11 (31)	9 (26)	0.793
CKD ^a^, *n* (%)	2 (29)	2 (29)	1.0	16 (44)	17 (49)	0.814
Anemia WHO, *n* (%)	3 (43)	2 (29)	1.0	23 (64)	21 (60)	0.809
Anemia, Hb < 12 g/dL, *n* (%)	0 (0)	0 (0)	1.0	16 (44)	13 (37)	0.631
Comorbidity index	3 (1)	3 (1)	1.0	4 (1)	3 (1)	0.266
**Treatments (%)**						
ACEI or ARBs	5 (71)	5 (71)	1.0	24 (67)	27 (77)	0.430
Beta-blockers	6 (86)	5 (71)	1.0	34 (94)	32 (91)	0.674
Diuretics	7 (100)	6 (86)	1.0	32 (89)	31 (89)	1.0
**Laboratory**						
Hemoglobin, g/dL	13.6 (1.2)	13.4 (0.6)	0.645	11.9 (1.5)	12.2 (1.3)	0.321
eGFR, ml/min/1.73 m^2^	65 (21)	65 (19)	0.975	67 (28)	65 (33)	0.839
NT-proBNP, pg/mL	1574 (497–1028)	536 (481–1178)	0.318	1892 (832–4776)	1450 (536–5901)	0.890
hs-CRP	0.4 (0.2–1.4)	0.5 (0.2–6.2)	0.628	0.6 (0.3–1.4)	0.3 (0.2–0.9)	0.086
**Iron status and hematinic**						
Serum iron	50.9 (9.2)	85.3 (29.3)	0.012	45.8 (17.2)	89.6 (32.8)	<0.001
Serum ferritin	57.3 (32.2)	262.0 (132.8)	0.002	45.4 (26.9)	322.9 (238.8)	<0.001
TSAT	13.2 (2.0)	25.4 (6.3)	<0.001	11.4 (3.6)	27.1 (10.7)	<0.001
sTfR	2.1 (0.4)	1.5 (0.8)	0.095	2.0 (0.6)	1.2 (0.4)	<0.001

Data presented as mean ± SD, *N* (%) or median (interquartile range). ACEI (angiotensin converting enzyme inhibitor), ARB (angiotensin receptor blocker), BMI (body mass index), BP (blood pressure), CKD (chronic kidney disease), HF (heart failure), Hb (Hemoglobin), COPD (chronic obstructive pulmonary disease), eGFR (estimated glomerular filtration rate), hs-CPR (high sensitive reactive c protein), LVEF (left ventricular ejection fraction), MRA (mineralocorticoid receptor antagonist), NT-proBNP (N-terminal pro-B type natriuretic peptide), NYHA (New York Heart Association), sTfR (serum soluble transferrin receptor), TSAT (transferrin saturation), WHO (world health organization). ^a^ CKD was defined as eGFR < 60 (MDRD).

**Table 2:** General function of the 22 candidate genes analyzed by TLDA (TaqMan^®^ Low Density Array) in cohort #2.

Gene Name	Symbol	Function
Aconitase 1	ACO1	Essential enzyme in the TCA cycle and interacts with mRNA to control de levels of iron inside cells
Adrenomedullin	ADM	Vasodilation, regulation of hormone secretion, promotion of angiogenesis and antimicrobial activity
ATPase H+ transporting accessory protein 2	ATP6AP2	Associated with ATPases (fundamental roles in energy conservation, secondary active transport…)
Cytochrome C oxidase assembly factor 3	COA3	Localized to mitochondria and essential for cytochrome c oxidase function. Component of MITRAC
Cytochrome C oxidase copper chaperone 17	COX17	Copper metallochaperone essential for the assembly of the mitochondrial respiratory chain complex IV (cytochrome c oxidase)
C-X-C motif chemokine ligand 8	CXCL8	Member of C-X-C chemokine family and is a major mediator of the inflammation response
Endothelin-converting enzyme 1	ECE1	Proteolytic processing of endothelin precursors to biologically active peptides
Mitochondrial Ferritin	FTMT	Stores iron in a soluble, non-toxic, readily available form (ferric iron binding) and ferroxidase activity
Hydroxyacyl-coA dehydrogenase trifunctional multienzyme complex subunit alpha	HADHA	Subunit of the mitochondrial protein which catalyzes the last 3 steps of mitochondrial beta-oxidation of long chain fatty acids
Hypoxia inducible domain family member 1A	HIGD1A	Subunit of cytochrome c oxidase, may play a role in the assembly of respiratory super complexes in mitochondria
Hemopexin	HPX	Plasma glycoprotein that binds heme with high affinity and may be involved in protecting cells from oxidative stress
Lipocalin-2	LCN2	Iron-trafficking protein involved in multiple processes (apoptosis, innate immunity and renal development)
DNA Ligase 3	LIG3	Member of the DNA ligase family, involved in excision repair and is in mitochondria and nucleus
Mitochondrial calcium uniporter regulator 1	MCUR1	Key regulator of MCU (Mitochondrial calcium uniporter) required for calcium entry into mitochondria
Metallothionein 2A	MT2A	Member of the metallothionein family, act as anti-oxidant, important in homeostatic control of metal in cell
Myosin heavy chain 7B	MYH7B	Encodes a heavy chain of myosin II, involved in muscle contraction
Sirtuin 7	SIRT7	NAD-dependent protein-lysine deacylase; functions of huma sirtuins have not yet been well determined
Small integral membrane protein 20	SMIM20	Component of MITRAC complex, that regulates cytochrome c oxidase assembly in mitochondria
STEAP3 metalloreductase	STEAP3	Multipass membrane protein that functions as an iron transporter
Transferrin Receptor	TFRC	Cell surface receptor necessary for cellular iron uptake by receptor-mediated endocytosis
ATP-dependent Zinc metalloprotease	YME1L1	ATP-dependent metalloprotease that catalyzes the degradation of folded and unfolded proteins in mitochondria
Zinc finger protein 260	ZNF260	Transcription factor that acts as a cardiac regulator and an effector of alpha1-adrenergic signaling

**Table 3:** Fold-change of candidate genes in a chronic HF population with systemic ID versus no-ID.

Symbol	ID vs. Ct (Fold Change)	*p*-Value
ACO1	0.76	0.18
ADM	0.62	0.03
ATP6AP2	0.98	0.87
COA3	0.85	0.42
COX17	0.66	0.08
CXCL8	1.18	0.53
ECE1	0.70	0.03
FTMT	0.26	0.02
HADHA	0.95	0.74
HIGD1A	1.01	0.95
HPX	1.04	0.85
LCN2	0.86	0.36
LIG3	0.71	0.09
MCUR1	0.76	0.15
MT2A	1.05	0.79
MYH7B	0.87	0.56
SIRT7	0.63	0.02
SMIM20	0.68	0.04
STEAP3	1.02	0.92
TFRC	1.31	0.03
YME1L1	0.82	0.28
ZNF260	0.51	0.13

**Table 4:** Adjusted Cox proportional hazards analyses exploring associations of gene expression with outcomes.

	**Composite Primary Endpoint**		
	**HR**	**95% CI**	***p*-Value**
FTMT Δct, T2 vs. T1 + T3	2.396	1.043–5.502	0.039
SIRT71 Δct, T2 + T3 vs. T1	5.495	1.784–16.923	0.003
SMIM20 Δct, T2 + T3 vs. T1	9.511	2.698–33.530	0.000459
TFRC Δct, T3 vs. T1 + T2	1.042	0.347–3.126	0.942
ADM Δct, T2 + T3 vs. T1	1.296	0.456–3.683	0.626
ECE1 Δct, T2 + T3 vs. T1	1.489	0.588–3.767	0.401
	**All-Cause Death**		
	**HR**	**95% CI**	***p*-Value**
FTMT Δct, T2 vs. T1 + T3	4.448	1.497–13.216	0.007
SIRT71 Δct, T2 + T3 vs. T1	7.122	1.848–27.438	0.004
SMIM20 Δct, T2 + T3 vs. T1	7.44	1.882–27.930	0.03
TFRC Δct, T3 vs. T1 + T2	2.056	0.700–6.036	0.19
ADM Δct, T2 + T3 vs. T1	1.497	0.460–4.869	0.502
ECE1 Δct, T2 + T3 vs. T1	1.083	0.310–3.783	0.9

Adjusted Cox proportional hazards models using backwards methods. Δct (change in change in gene expression cycle threshold) gene expression tertiles according to generalized additive models of mRNA levels: T1 (lower expression), T2 (intermediate expression), T3 (higher expression). ADM (adrenomedullin), ECE1 (endothelin converting enzyme 1), FTMT (mitochondrial ferritin), TFRC (transferrin receptor), SMIM20 (small integral membrane protein 10), SIRT7 (sirtuin-7).

## Data Availability

All relevant data are included in this published article.

## Supplementary Materials

The following are available online at https://www.mdpi.com/article/10.3390/jcm10214937/s1. Supplementary Figure S1: Enriched gene ontology (GO) analysis of differential expression genes observed in the transcriptome analyses of blood samples in cohort #1. Supplementary Figure S2: Smooth spline estimates of all-cause death according to gene expression. Supplementary Figure S3: Adjusted survival curves of significant associations between gene expression and primary composite endpoint (all-cause death or HF hospitalization). Supplementary Figure S4. Adjusted survival curves of significant associations between gene ex-pression and all-cause death.
