# Supplementary material for: Blood Differential Gene Expression in Patients with Chronic Heart Failure and Systemic Iron Deficiency: Pathways Involved in Pathophysiology and Impact on Clinical Outcomes
Source: J Clin Med. 2021 Oct 26;10(21):4937. doi: 10.3390/jcm10214937 (PMC8585093; doi:10.3390/jcm10214937)
Supplement: Supplementary file 1 [file jcm-10-04937-s001.zip › jcm-1394128-supplementary.pdf]

Figure S1: Enriched gene ontology (GO) analysis of differential expression genes observed in the transcriptome analyses of blood samples in cohort #1. Analysis was carried out by Metascape. The x-axis denotes –log10 (P) values based on the cumulative hypergeometric distributions. The colors denote de relative value of –log10 (P): darker colors indicate a greater value of –log10 (P).

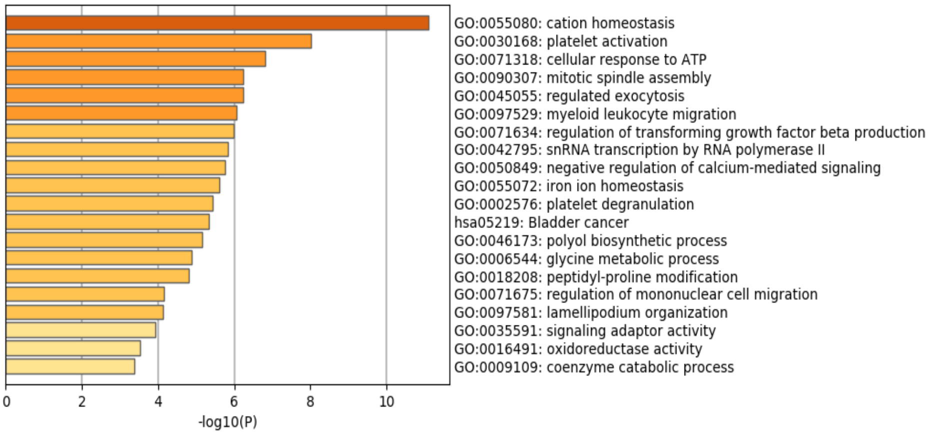

Supplementary Figure S2: Smooth spline estimates of all-cause death according to gene expression of *ADM* (A), *ECE1* (B), *FTMT* (C), *SIRT7* (D), *SMIM20* (E) and *TFRC* (F). Δct: change in gene expression cycle threshold). Ct levels are inversely proportional to the amount of target nucleic acid in the sample (i.e., the lower the Ct level the greater the amount of target nucleic acid in the sample). Plots show the beta estimates of the outcome.

(A)

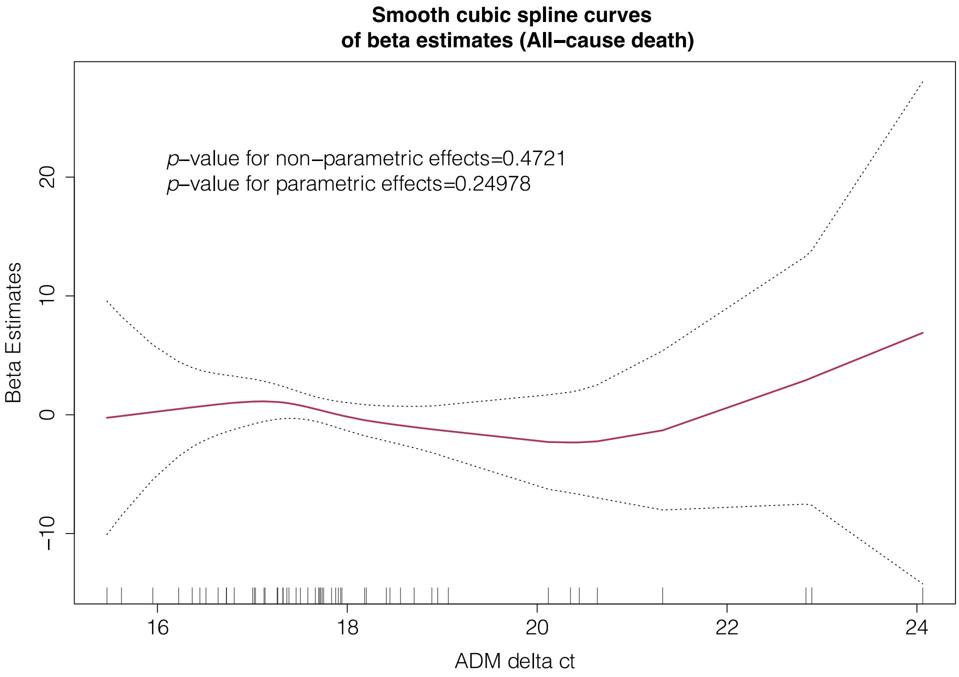

(B)

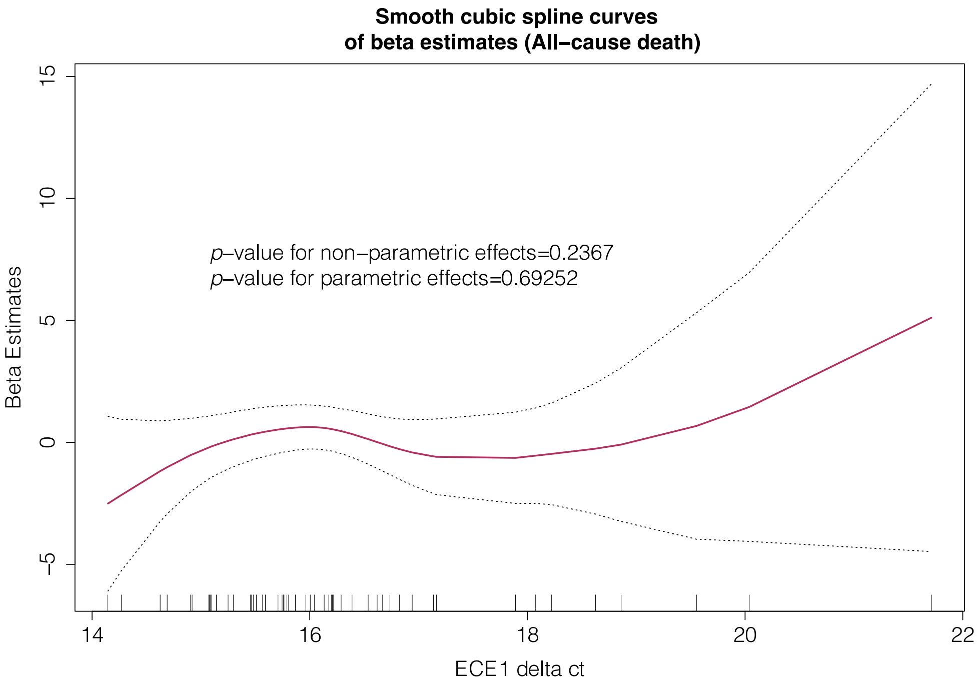
Smooth cubic spline curves
of beta estimates (All−cause death)
p−value for non−parametric effects=0.2367
p−value for parametric effects=0.69252
Beta Estimates
ECE1 delta ct
−5
0
5
10
15
14
16
18
20
22

(C)

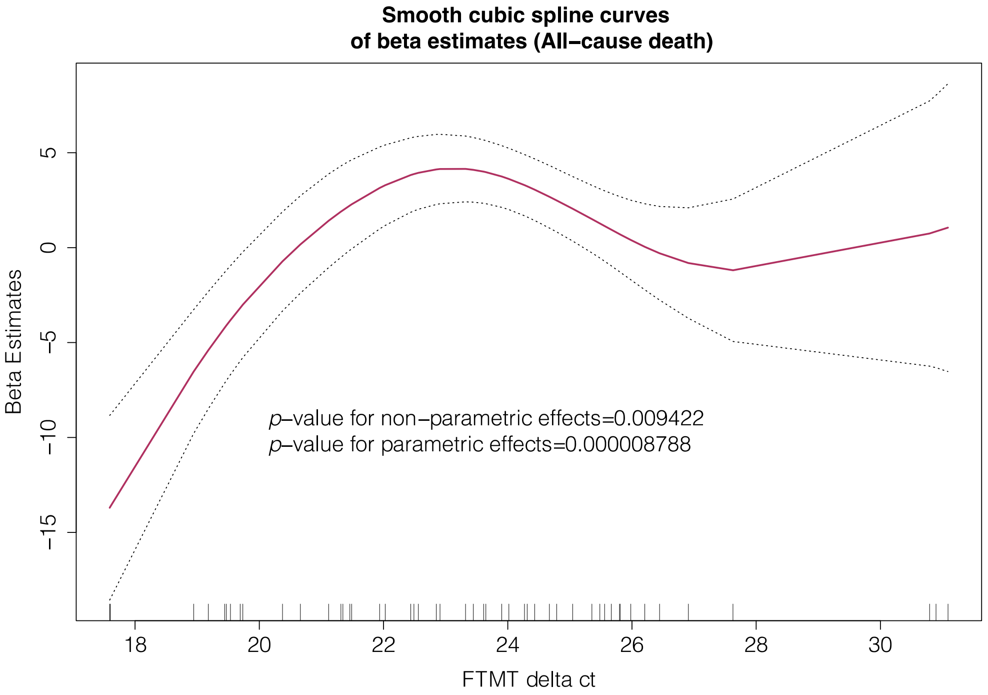
Smooth cubic spline curves
of beta estimates (All−cause death)
p−value for non−parametric effects=0.009422
p−value for parametric effects=0.000008788
Beta Estimates
FTMT delta ct
−15
−10
−5
0
5
18
20
22
24
26
28
30

(D)

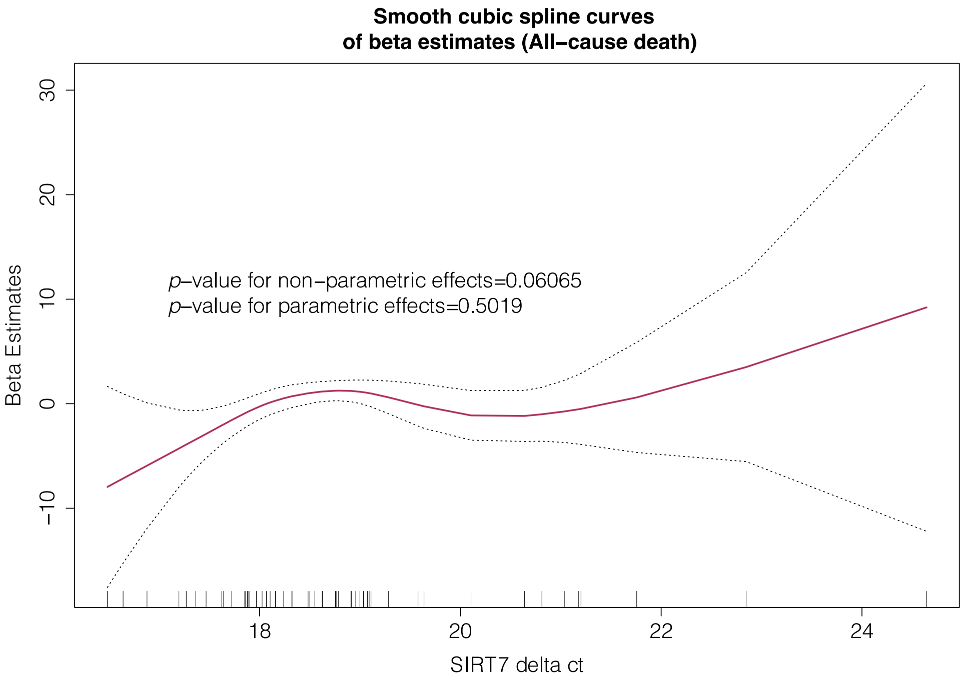
Smooth cubic spline curves
of beta estimates (All−cause death)
p−value for non−parametric effects=0.06065
p−value for parametric effects=0.5019
Beta Estimates
30
20
10
0
−10
18
20
22
24
SIRT7 delta ct

(E)

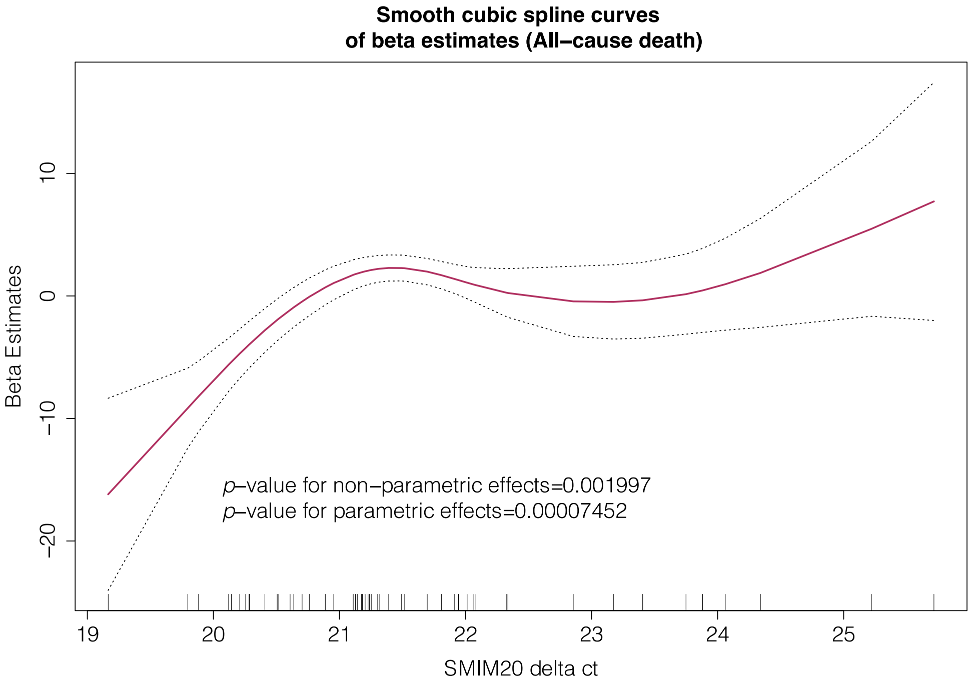
Smooth cubic spline curves
of beta estimates (All−cause death)
p−value for non−parametric effects=0.001997
p−value for parametric effects=0.00007452
Beta Estimates
10
0
−10
−20
19
20
21
22
23
24
25
SMIM20 delta ct

(F)

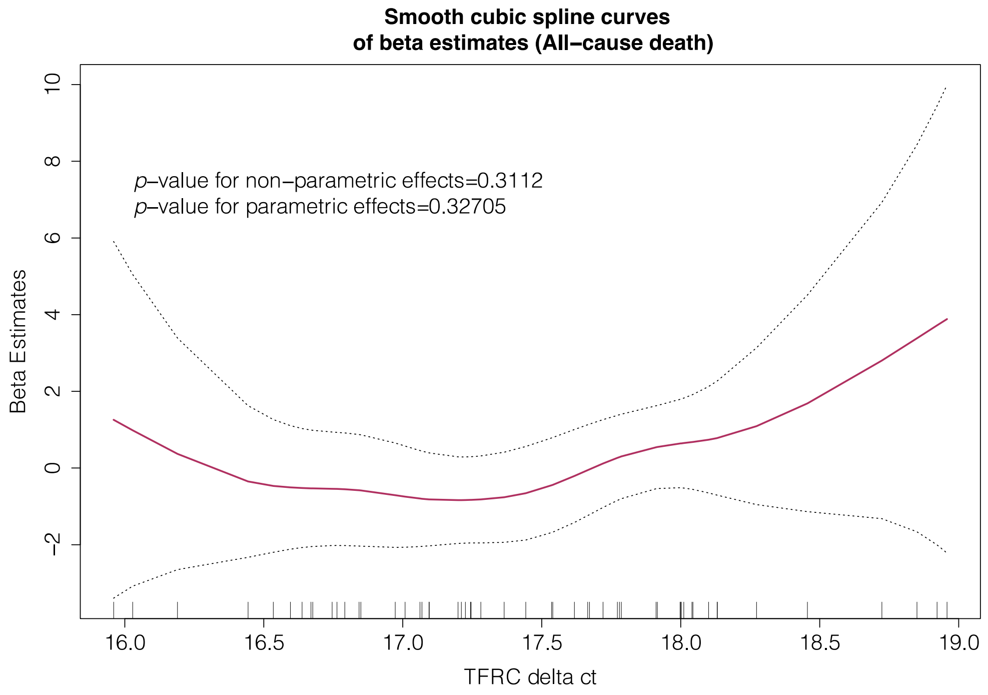

Supplementary Figure S3: Adjusted survival curves of significant associations between gene expression and primary composite endpoint (all-cause death or HF hospitalization). Δct express change in gene expression cycle threshold.

Panel A: FTMT mid-range expression (tertile 2 of Δct) compared to high expression (lower tertile of Δct –tertile 1) or low expression (higher Δct –tertile 3) of FTMT.

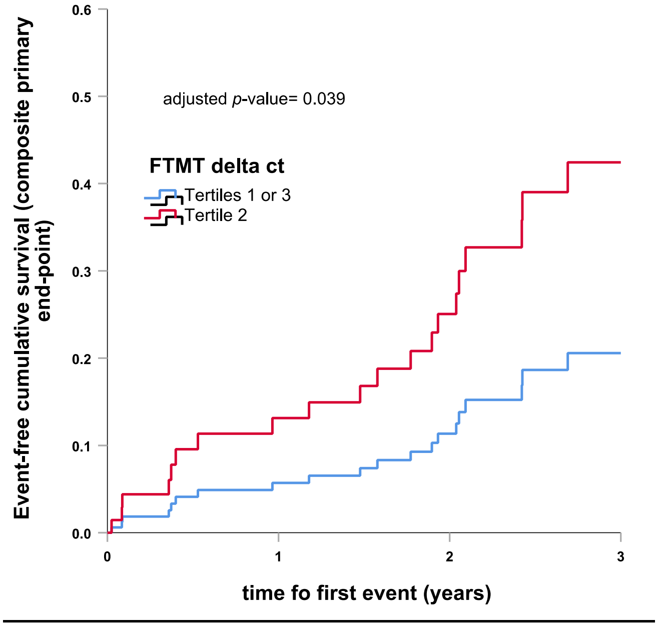

Panel B: SIRT7 mid-range expression (tertile 2 of Δct) or low expression (higher Δct –tertile 3) compared to high expression (lower tertile of Δct –tertile 1) of SIRT7.

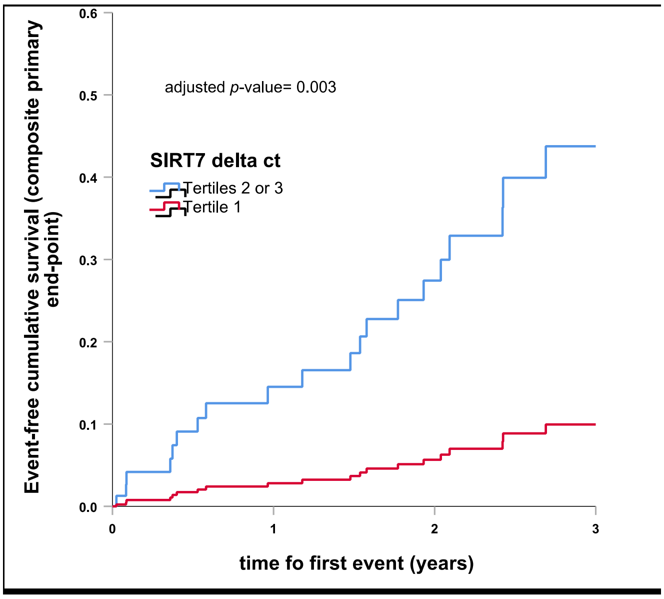

Panel C: SMIM20 mid-range expression (tertile 2 of Δct) or low expression (higher Δct –tertile 3) compared to high expression (lower tertile of Δct –tertile 1) of SMIM20.

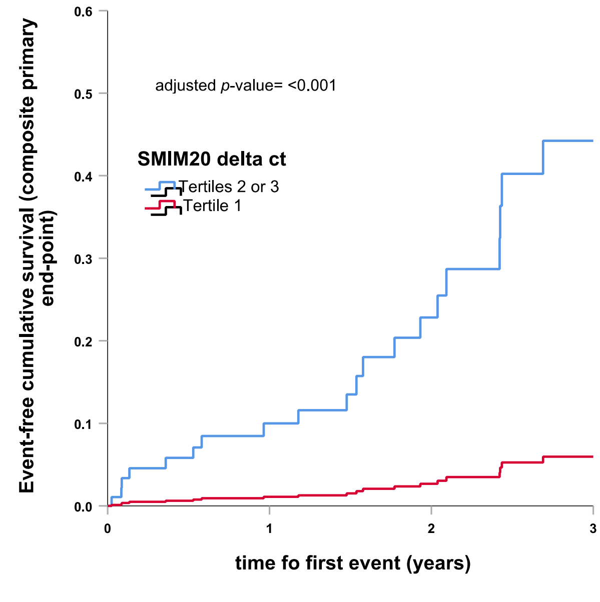

Supplementary Figure S4. Adjusted survival curves of significant associations between gene expression and all-cause death. Δct: change in cycle threshold gene expression

Panel A: FTMT mid-range expression (tertile 2 of Δct) compared to high expression (lower tertile of Δct –tertile 1) or low expression (higher Δct –tertile 3) of FTMT.

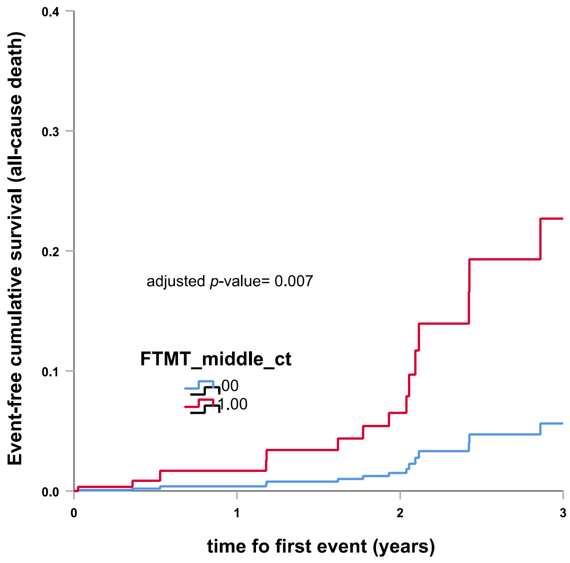

Panel B: SIRT7 mid-range expression (tertile 2 of Δct) or low expression (higher Δct –tertile 3) compared to high expression (lower tertile of Δct –tertile 1) of SIRT7.

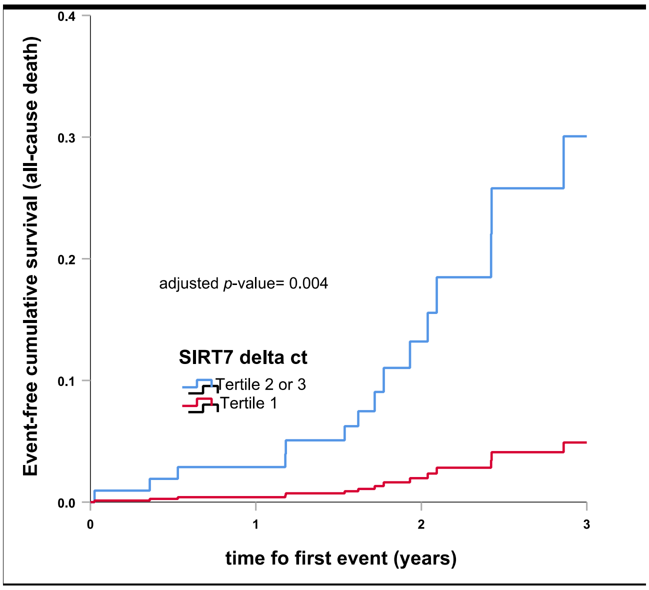

Panel C: SMIM20 mid-range expression (tertile 2 of Δct) or low expression (higher Δct –tertile 3) compared to high expression (lower tertile of Δct –tertile 1) of SMIM20.

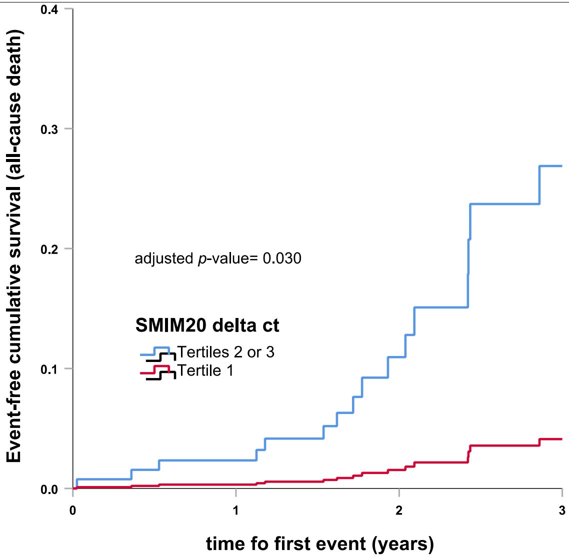
Event-free cumulative survival (all-cause death)
0.4
0.3
0.2
0.1
0.0
adjusted p-value= 0.030
SMIM20 delta ct
Tertiles 2 or 3
Tertile 1
0
1
2
3
time fo first event (years)
